# PHI density prospectively improves prostate cancer detection

**DOI:** 10.1007/s00345-020-03585-2

**Published:** 2021-01-20

**Authors:** Carsten Stephan, Klaus Jung, Michael Lein, Hannah Rochow, Frank Friedersdorff, Andreas Maxeiner

**Affiliations:** 1 Department of Urology, Charité-Universitätsmedizin Berlin, corporate member of Freie Universität Berlin, Charitéplatz 1, 10117 Berlin, Germany; 2 Berlin Institute for Urologic Research, Berlin, Germany; 3 Department of Urology, Sana Hospital, Offenbach, Germany

**Keywords:** Prostate health index (PHI), PHI density (PHID), Prostate cancer, Decision curve analysis (DCA)

## Abstract

**Purpose:**

To evaluate the Prostate Health Index (PHI) density (PHID) in direct comparison with PHI in a prospective large cohort.

**Methods:**

PHID values were calculated from prostate-specific antigen (PSA), free PSA and [− 2]proPSA and prostate volume. The 1057 patients included 552 men with prostate cancer (PCa) and 505 with no evidence of malignancy (NEM). In detail, 562 patients were biopsied at the Charité Hospital Berlin and 495 patients at the Sana Hospital Offenbach. All patients received systematic or magnetic resonance imaging (MRI)/ultrasound fusion-guided biopsies. The diagnostic accuracy was evaluated by receiver operating characteristic (ROC) curves comparing areas under the ROC-curves (AUC). The decision curve analysis (DCA) was performed with the MATLAB Neural Network Toolbox.

**Results:**

PHID provided a significant larger AUC than PHI (0.835 vs. 0.801; *p* = 0.0013) in our prospective cohort of 1057 men from 2 centers. The DCA had a maximum net benefit of ~ 5% for PHID vs. PHI between 35 and 65% threshold probability. In those 698 men within the WHO-calibrated PSA grey-zone up to 8 ng/ml, PHID was also significantly better than PHI (AUC 0.819 vs. 0.789; *p* = 0.0219). But PHID was not different from PHI in the detection of significant PCa.

**Conclusions:**

Based on ROC analysis and DCA, PHID had an advantage in comparison with PHI alone to detect any PCa but PHI and PHID performed equal in detecting significant PCa.

## Introduction

Prostate-specific antigen (PSA) has a low specificity in the detection of prostate cancer (PCa). In 2010, the Prostate Health Index PHI ([− 2]proPSA/freePSA × √PSA) was implemented [1]. In 2012, the FDA approved PHI for PCa detection and PHI has been established as PCa biomarker [2]. Different multicenter studies [3–5] showed improvement in comparison with percent free PSA (%fPSA). PHI also identifies clinical significant PCa [6]. PHI further correlates with tumor volume [7] and can predict pathological outcome [8] and tumor recurrence [9] in radical prostatectomy patients. In 2014, the term PHI density (PHID: PHI/prostate volume) was proposed in a study on 275 men including 189 men with no evidence of malignancy (NEM) and 86 PCa patients [10]. The difference in the area under the receiver operating characteristic (ROC)-curve (AUC) between PHI (0.76) and PHID (0.77) was small and multivariate models only marginally improved AUCs to 0.79 [10]. Since then, only few PHID studies were published [11, 12]. Tosoian et al. [12] calculated the largest AUC for PHID with 0.84 while PHI and %fPSA had 0.76 and 0.75, respectively. Contrarily, Friedl et al. [11] found a higher AUC for PHI (0.79) than PHID (0.77). Both studies include a relatively low patient number with 112 [11] and 118 men [12].

Aims of this study were:

(i) to investigate the value of PHID to detect any PCa in comparison with PHI in a large prospective cohort with > 1000 men and
(ii) to test the diagnostic capacity of PHID in different subgroups and for the detection of clinically significant PCa with Gleason score ≥ 7.

## Materials and methods

Based on former patient data from 2002 to 2014 with PSA, free PSA and [− 2]proPSA and prostate volume from three surveys [5, 13, 14], we initiated in 2014 a prospective collection.

Our prospective group consisted of 1057 men with 552 PCa patients (52.2%) and 505 NEM patients. Between 2014 and 2019, patients were consecutive biopsied within two tertiary hospitals: 562 patients at the Charité Hospital Berlin and 495 patients at the Sana Hospital Offenbach. Magnetic resonance imaging (MRI)/ultrasound fusion-guided biopsies were performed only in Berlin (*n* = 52 of 562, 9.3%). Prostate volume was determined by transrectal ultrasound. Detailed exclusion criteria such as prostatitis or others have been applied as described before [5, 14]. The respective hospital ethics committee approved the study. All patients provided written informed consent. Histological results were related to the 2014 proposed ISUP Gleason grading system. A Gleason score ≥ 7 PCa was defined as clinically significant PCa.

Serum samples were prospectively collected and always frozen at − 80 °C until analysis. The PSA ranged from 0.49 to 25.8 ng/ml and the PSA calibration was performed based on the WHO PSA reference material. The fully automated immunoassay device Access^®^ (Beckman Coulter, Brea, California) was used for all samples. Measurements of PSA, free PSA and [− 2]proPSA were performed in Berlin.

### Statistical analysis

The MedCalc version 15.8 (MedCalc Software, Ostend, Belgium) was used for statistical analysis and ROC analysis. Group differences were assessed by the nonparametric Mann–Whitney *U* test and correlations were analyzed using the Spearman rank correlation coefficient (*r*_s_).

Decision curve analysis (DCA) was performed with the MATLAB Neural Network Toolbox (Mathworks) as described earlier [15]. In the DCA, a possible benefit of a marker or model is plotted against threshold probabilities, which then yields the decision curve. The DCA can identify the range of threshold probabilities and the magnitude of benefit, where the marker or model is of value. Two-sided *p* values < 0.05 were considered statistically significant.

## Results

The patient characteristic of the cohort is provided in Table 1. All tested parameters differed significantly between both groups, PCa and NEM, respectively. Both, PHI (*r*_s_ = 0.38, confidence interval CI 0.31–0.45) and PHID (*r*_s_ = 0.30, CI 0.23–0.38) correlated significantly (*p* < 0.0001) with the Gleason score.

**Table 1:** Clinical characteristics (medians and interquartile ranges) of the study cohort

Parameter (medians)	All patients (*n* = 1057)	PCa (*n* = 552)	NEM (*n* = 505)	*p* value
Age (years)	69 (62–73)	67 (61–72)	70 (64–75)	< 0.0001
Prostate volume (cm^3^)	47 (34–66)	39 (29–52)	59 (44–80)	< 0.0001
PSA (ng/ml)	6.41 (4.33–9.24)	6.68 (4.74–9.36)	6.02 (3.74–9.12)	0.0006
%fPSA (%)	14.9 (10.5–21.5)	12.0 (8.84–16.5)	18.9 (14.2–25.3)	< 0.0001
PSAD	0.135 (0.09–0.21)	0.17 (0.11–0.26)	0.1 (0.06–0.15)	< 0.0001
PHI	52.3 (38.1–72.5)	65.3 (50.2–87.7)	40.4 (30.6–53.9)	< 0.0001
PHID	1.1 (0.62–1.98)	1.73 (1.12–2.61)	0.66 (0.44–1.01)	< 0.0001

*PSA* prostate-specific antigen, *%fPSA* percent free PSA, *PSAD* PSA density, *PHI* Prostate Health Index, *PHID* PHI density

**Fig. 1:**
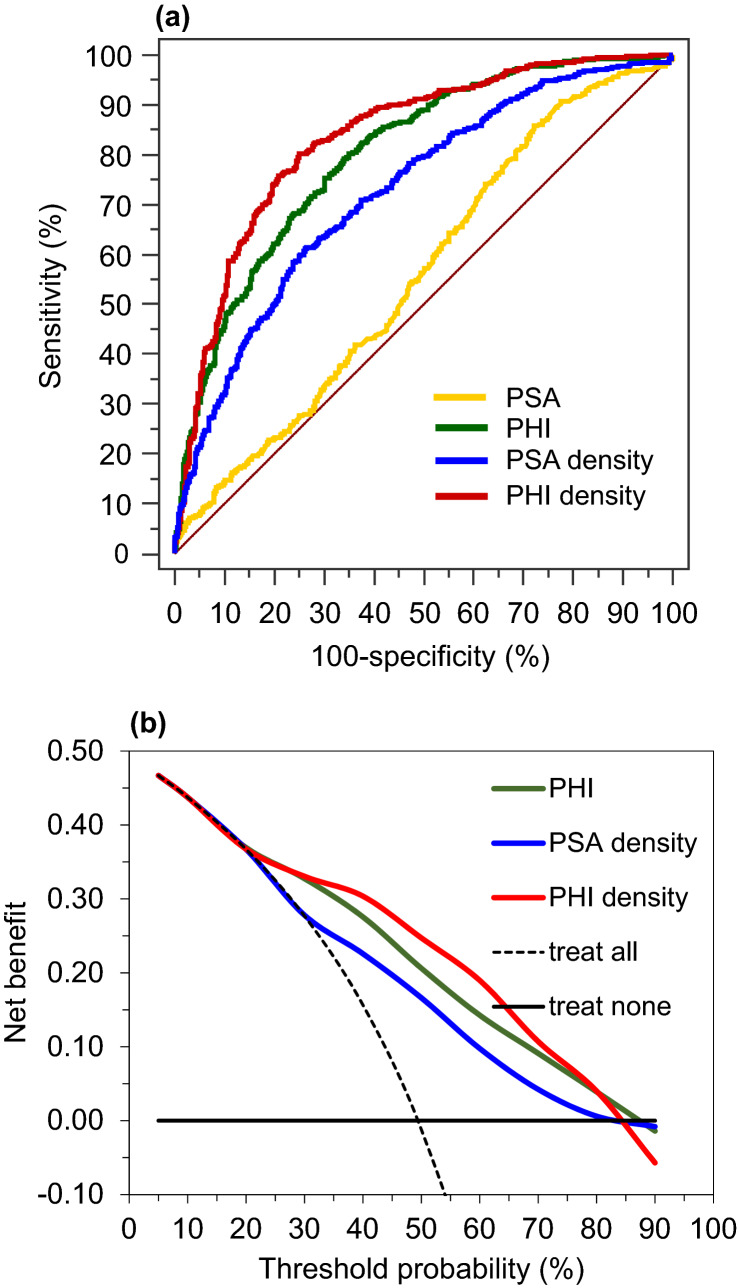
The prospective group with 1057 patients with **a** receiver operating characteristic for PSA (AUC: 0.561), PSAD (0.726), PHI (0.801) and PHID (0.835) and with **b** decision curve analysis (DCA) comparing model 1 using PHI with model 2 using PSAD with model 3 using PHID, to biopsy-all and biopsy-none strategies

The AUC for PHID (0.835) was significantly larger than the AUC for PHI (0.801, *p* = 0.0013) (Fig. 1a). PHID outperformed all other parameters including PSA (AUC 0.561), PSA density (PSAD, AUC 0.726) and %fPSA (AUC 0.753, all *p* < 0.0001). Additionally, the DCA showed an advantage of maximum 4–5% net benefit for PHID for a broad range between 35 and 65% threshold probability (Fig. 1b).

The Hybritech-calibrated PSA grey-zone of 2–10 ng/ml corresponds to WHO-calibrated values up to 8 ng/ml. A biopsy decision within this specific PSA range is mostly difficult. We therefore additionally analyzed those 698 men with PSA values 1–8 ng/ml. PHID was significantly better than PHI, but the AUC difference was again only 0.03 (0.819 vs. 0.789; *p* = 0.0219) (Table 2). In comparison to all patients, the AUCs for PSAD and %fPSA were only slightly below the AUC of PHI.

**Table 2:** AUC comparison of the cohort with PSA values 1–8 ng/ml in *n* = 698 patients

Parameter	AUC	95% confidence interval	*p* value versus PHI
PSA	0.591	0.55–0.63	< 0.0001
%fPSA	0.748	0.71–0.78	0.0377
PSAD	0.749	0.72–0.78	0.0361
PHI	0.789	0.76–0.82	–
PHID	0.819	0.79–0.85	0.0219

*AUC* area under the ROC curve*, PSA* prostate-specific antigen, *%fPSA* percent free PSA, *PSAD* PSA density, *PHI* Prostate Health Index, *PHID* PHI density

A distribution of NEM (*n* = 505) and low risk patients (Gleason score < 7, *n* = 87) combined (*n* = 505 + 87 = 593) vs. all other PCa (Gleason score ≥ 7, *n* = 465) provided no further improvement of PHID (AUC 0.786) in comparison with PHI (AUC 0.792, *p* = 0.62). When comparing only the 87 low risk PCa patients (Gleason score < 7) with all other 465 PCa (Gleason score ≥ 7), the AUCs for PHID (0.715) and PHI (0.74, *p* = 0.27) did again not differ from each other.

Different prostate volume cutoffs were also evaluated but no improvements for PHID were found. For those 408 patients with small volumes ≤ 40 cm^3^, PHI and PHID were not significantly different between PCa and NEM (AUCs: 0.729 vs. 0.721, *p* = 0.58). Within this group with prostate volume ≤ 40 cm^3^ the Gleason score ≤ 6 PCa (*n* = 38) as well as the NEM (*n* = 99) together (*n* = 137) provided also no difference in comparison with the Gleason score ≥ 7 PCa (*n* = 271) regarding PHI and PHID (AUCs: 0.74 vs. 0.736, *p* = 0.77). The same was visible when comparing the 38 low risk PCa patients (Gleason score < 7) with the 271 Gleason score ≥ 7 PCa (AUCs: 0.74 vs. 0.736, *p* = 0.77). The remaining 649 patients (volume > 40 cm^3^) also showed no difference between PHID (AUC: 0.82) and PHI (0.802, *p* = 0.15).

## Discussion

Biomarkers for PCa detection play an important role and PHI is able to further improve specificity over PSA and %fPSA [16, 17]. While one survey in 112 men found a higher AUC for PHI (0.79) than for PHID (0.77) [11], others showed in 275 and 118 men a further improvement when using PHID (0.77 or 0.84) instead of PHI only (each 0.76) [10, 12].

The results of our large prospective cohort confirm a further advantage using PHID in comparison with PHI. The absolute AUC difference between 0.8 for PHI and 0.835 for PHID in our group (Fig. 1a) is small but significant. The same AUC advantage for PHID (0.819) vs. PHI (0.789) is visible within the PSA grey-zone 1–8 ng/ml (Table 2). More importantly, the DCA revealed an identical advantage for PHID between 35 and 65% threshold probability (Fig. 1b) in the whole cohort and within the PSA grey-zone (data not shown). The importance of a DCA net benefit in relation to an improved AUC has been discussed elsewhere [18].

Our data are in line with the results of Tosoian et al. [12], where the authors claimed in respect to their 118 patients, that PHID has the strongest discriminative ability for clinically significant PCa with an AUC of 0.84. In our significantly larger study group with 1057 patients, PHID had a similar AUC of 0.835. Thus, PHID currently represents the best discriminative value for PCa detection. However, PHI alone did not differ from PHID in any AUC comparison with different volume cutoffs nor in detecting significant Gleason score ≥ 7 PCa. This shows the excellent discriminatory power of PHI independently from prostate volume, which has been confirmed in a recent study in more than 1600 Asian men [19]. Our data confirmed the earlier described phenomenon by Friedl et al. [11], where the AUC for PHI (0.79) was reported higher than the AUC of PHID (0.77). A subgroup analysis within our study based on various prostate volume cutoffs found always similar AUCs for PHI in comparison with PHID without statistical differences. Because PHID includes prostate volume, subgroup analyses with either selected small or relatively large glands might be responsible for somewhat lower AUCs for PHID in comparison with PHI. PHI might also be the preferred parameter in small glands ≤ 40 cm^3^ because fPSA as part of the PHI formula showed a better discriminatory power in these patients [20]. Additionally, a smaller number of patients (*n* = 112) in the mentioned study [11] might also influence results in favor for PHI despite this phenomenon has been recently also partially described in a large cohort [21].

A comparison of PHI with another current prostate biomarker, the four-kallikrein panel showed comparable results [22]. However, there is a difference for a possible routine use between the FDA approved PHI with its availability in hospitals and reference laboratories and the four-kallikrein panel, which is not approved and only available as a send-out to the company. An assessment of the four-kallikrein panel together with prostate volume in a large group is still lacking.

PHID (*r*_s_ = 0.30) further showed a weaker but still significant correlation to Gleason score in comparison with PHI (*r*_s_ = 0.38). This is in congruence with initial %[− 2]proPSA density (*r*_s_ = 0.205, *p* = 0.05) and PHI (*r*_s_ = 0.22, *p* = 0.039) data, where the density value correlated also weaker than PHI with the Gleason score [10].

Conformingly, PHID could not improve the detection of clinically significant PCa with Gleason score ≥ 7 in comparison with NEM and Gleason score < 7 combined in our prospective cohort. This phenomenon with no improvement for clinically significant PCa has been earlier described for PHI in smaller cohorts, too [23, 24]. PHI was also not able to detect significant PCa with Gleason score ≥ 7 [23, 24]. Contrary, in earlier large studies PHI could preferentially detect aggressive PCa [3–5, 25]. A recent nomogram using PHI and prostate volume also detected aggressive PCa [26].

Druskin et al. [27] combined PHID with MRI and prior negative biopsy status in 241 patients for the diagnosis of clinically significant PCa. Their PHID medians were 1.18 and 0.55 in men with and without clinically significant PCa [27]. These absolute PHID medians are different from our 698 patients (356 PCa and 342 NEM) with PSA values < 8 ng/ml with 1.63 for any PCa and 0.67 for NEM. Possible reasons for our higher values might be no prior biopsies and no MRI fusion biopsies. The inclusion of MRI data with the Prostate Imaging Reporting and Data System (PI-RADS) score into multivariable models seems to be reasonable and potentially further enables PCa diagnostics with an AUC increase from 0.78 (all 241 men) to 0.90 (subgroup of 104 men with MRI) for PHID [27]. In 2014, Porpiglia et al. [28] first combined MRI, PHI and PCA3 in 170 patients. They reported very high AUC values > 0.9 but the PI-RADS scoring was not used [28]. Currently, most patients already present a suspicious MRI for biopsy decision. PHI and PHID should be used in cases of a not suspicious MRI and continuously elevated PSA values. Only when MRI and PHI are not suspicious, a biopsy should be avoided.

Recently, a study by Lopes Vendrami et al. [29] on 211 men with PHID results and at least one suspected MRI lesion PI-RADS ≥ 3 was published. PHI and PHID showed comparable AUCs of 0.78 and 0.82 with our data and the authors further concluded that the use of MRI/ultrasound fusion-guided biopsies in comparison to systematic biopsies may have favored the results for PHID [29]. Most recently, Hsieh et al. [30] combined not PHID but PHI with MRI results in 102 men and they found an improved AUC from 0.735 (only PHI) to 0.873. As a weakness of our study, we had only 52 patients with fusion biopsies. Despite neither PHI (48.5) nor PHID (1.01) were different from those patients without fusion biopsies (46.3 and 0.94), further recommendations based on our data cannot be given. As further limitation, we did not collect information on family history on PCa and we did also not include the digital rectal exam into analysis.

Finally, this prospective study with more than 1000 patients confirms the initial hypothesis from 2014, that prostate dimension-adjusted PSA subforms may better differentiate between PCa and NEM patients and that especially PHID offers a gain in accuracy with respect to PSA, PSAD, %fPSA and PHI [10]. While Mearini et al. [10] found in their prospective group of 275 men with PSA values of 2–10 ng/ml including 26 PCa patients with Gleason score ≥ 7 an AUC gain between 0.05 and 0.08 in six different models to detect significant PCa, we could not confirm this advantage. In our much larger cohort with 84% (*n* = 465) Gleason score ≥ 7 PCa patients no significant difference between PHID (AUC 0.786) and PHI alone (AUC 0.792, *p* = 0.62) was visible.

## Conclusions

Our data could confirm a significant advantage for PHID in comparison with PHI alone in detecting any PCa. But PHI alone also reaches a high discriminatory power with no difference from PHID in detecting significant PCa with Gleason score ≥ 7. However, in line with the most recent study [29], we also recommend using both, prostate volume and PHI due to an improved diagnostic efficacy in PCa detection with the combined value PHID.
